# Exploring barriers to access to care following the 2021 socio-political changes in Afghanistan: a qualitative study

**DOI:** 10.1186/s13031-024-00595-4

**Published:** 2024-04-24

**Authors:** Alessandro Lamberti-Castronuovo, Martina Valente, Francesca Bocchini, Monica Trentin, Michela Paschetto, Ghulam Ali Bahdori, Jan Agha Khadem, Mirza Sayed Nadeem, Mohammad Hanif Patmal, Mohammad Tawoos Alizai, Rossella Miccio, Luca Ragazzoni

**Affiliations:** 1 CRIMEDIM, Center for Research and Training in Disaster Medicine, Humanitarian Aid and Global Health, Università del Piemonte Orientale, Via Lanino 1, Novara, 28100 Italy; 2 Department for Sustainable Development and Ecological Transition, Università del Piemonte Orientale, Vercelli, 13100 Italy; 3 EMERGENCY NGO ONLUS, Milan, 20122 Italy; 4 Department of Translational Medicine, Università del Piemonte Orientale, Novara, 28100 Italy

**Keywords:** Afghanistan, Access to care, EMERGENCY NGO, Qualitative research, UHC, Universal health coverage

## Abstract

**Background:**

Following the change of government in August 2021, the social and economic landscape of Afghanistan deteriorated into an economic and humanitarian crisis. Afghans continue to struggle to access basic healthcare services, making Universal Health Coverage (UHC) in the country a major challenge. The aim of this study was to perform a qualitative investigation into the main access to care challenges in Afghanistan and whether these challenges have been influenced by the recent socio-political developments, by examining the perspectives of health professionals and hospital directors working in the country.

**Methods:**

Health professionals working in facilities run by an international non-government organisation, which has maintained continuous operations since 1999 and has become a key health reference point for the population, alongside the public health system, and hospital directors working in government hospitals were recruited to participate in an in-depth qualitative study using semi-structured interviews.

**Results:**

A total of 43 participants from ten provinces were interviewed in this study. Four issues were identified as critical barriers to achieving UHC in Afghanistan: (1) the lack of quality human resources; (2) the suboptimal management of chronic diseases and trauma; (3) the inaccessibility of necessary health services due to financial hardship; (4) the unequal accessibility of care for different demographic groups.

**Conclusions:**

Health professionals and hospital directors shed light on weaknesses in the Afghan health system highlighting chronic issues and issues that have deteriorated as a result of the 2021 socio-political changes. In order to improve access to care, future healthcare system reforms should consider the perspectives of Afghan professionals working in the country, who are in close contact with Afghan patients and communities.

**Supplementary Information:**

The online version contains supplementary material available at 10.1186/s13031-024-00595-4.

## Background

On August 15, 2021, the political, social and economic landscape of Afghanistan abruptly changed following the Taliban takeover of the government in Kabul. The change of government brought about a reduction of fighting and violence in the country, but the lack of international recognition of the *de facto* authorities has resulted in a freezing of the country’s assets and the withdrawal of several humanitarian organisations from the country. Additionally, international donors imposed sanctions on the Afghan central bank, crippling the country’s economy [1].

Due to problems associated with limited cash liquidity, increasing food and fuel prices [2], and the massive wave of unemployment that followed the departure of humanitarian organisations, more than 20 million Afghans are in need of humanitarian assistance [3]. Prior to August 2021 the majority of health delivery was carried out by international organizations using primarily donor funds. After the changing government, direct international aid was suspended, causing a health crisis. However, after 6 weeks UNICEF and the World Health Organization (WHO) stepped in to oversee service contracts to fund health facilities in order to avert further collapse. Nevertheless, the national health system remains severely underfunded [2].

The majority of Afghan households face hunger and food insecurity: 95% of the population (∼ 39 M people) are not able to eat enough calories to sustain their health and the number of people experiencing acute hunger has reached 23 million [2]. The 2022 Humanitarian Needs Overview states that more than 18 M Afghans are living with extreme health needs [4].

Political, social and economic changes risk hindering access to care and jeopardizing the progress made in the last two decades [5]. Data from a 2022 survey from John Hopkins University shows that out of 131 participating healthcare workers (HCWs), almost 44% reported their working conditions as having worsened since August 2021 [6]. The ban of women from the labour market and the massive brain drain have further compromised Afghanistan’s capacity to deal with the humanitarian crisis [7].

Those who suffer the greatest barriers to access to care are vulnerable groups, including women, ethnic minorities, people with disabilities, and hard-to-reach communities. A 2020 study surveying 990 women reported that 46.2% of respondents did not visit health facilities during pregnancy [8]. Ethnic minorities face discrimination in accessing basic public goods and have become the target of acts of violence [9]. Even when significant advancements in healthcare provision happened in the country, people with disabilities did not experience improvements in their ability to access care [10, 11]. Most remote areas lack basic services [12], which reflects a situation that was already dire before the change of government. In 2021, Afghanistan had a Universal Health Coverage (UHC) Service Coverage Index of 41, which positioned the country at the bottom of the global ranking [13], and in 2020 had a density of 2.5 physicians per 10.000 population, below that of neighboring countries and many other fragile settings [14].

Ensuring access to care implies taking steps towards UHC [15, 16]. Recent changes occurred in Afghanistan might challenge the pursuit of UHC [17]. Access to care depends on both health system-related factors (e.g., availability and quality of services) and on social factors (e.g., the ability to perceive, pay and reach care) [18]. A recent nationwide multi-sectoral needs assessment study reports that families facing barriers to accessing care reached 80% in 2022 [19]. Access to care, and consequently the pursuit of UHC, are undermined by the shortage of HCWs and by the presence of regional disparities in health care coverage, with doctors’ distribution being eight times higher in Kabul than in more peripheral areas [20].

While quantitative assessments can yield insights about how health coverage has changed over time, a qualitative methodology is needed to understand the complex factors influencing access to care. This qualitative study sought to unravel barriers to access to care in Afghanistan from the perspectives of HCWs and hospital directors, and if and how these barriers modified after the change of government in August 2021. Although Afghanistan has a unique social, political and economic context, the results of this study may be applicable to other fragile settings undergoing changes in their socio-political assets, including a decrease in active fighting and violence [21].

## Methods

This study performs a qualitative investigation into how Afghan HCWs and health directors perceive the challenges in access to care, with an emphasis on changes following the 2021 change of government.

The research team performed an in-depth qualitative study using semi-structured interviews. The methods section is reported in accordance with the Consolidated Criteria for Reporting Qualitative Research (COREQ) [22].

This study is part of a larger project focusing on access to care which was performed collaboratively by the EMERGENCY non-governmental organization (NGO) and CRIMEDIM - Center for Research and Training in Disaster Medicine, Humanitarian Aid and Global Health, Università del Piemonte Orientale. In Afghanistan, EMERGENCY NGO has maintained continuous operations since 1999, offering the population free, high-quality care. It is funded by international donations and currently runs three tertiary hospitals (i.e., surgical, pediatric, maternity centres) as well as a network of first aid posts (FAP) and PHC clinics across 11 Afghan provinces.

### Study design

Purposive sampling was employed to select participants who could provide diversified data in terms of geographical distribution, gender, professional role. Participants had to be either HCWs working in facilities run by EMERGENCY NGO or hospital directors at the district, provincial or national level working for the Ministry of Public Health (MoPH). Upon request, interviewees received the interview guide in advance to decide whether or not to participate in the interview.

### Research team reflexivity

The data collection was carried out by a transdisciplinary research team: two researchers with a global health background and experience in qualitative research (ALC, MV), and the advocacy manager of the EMERGENCY NGO (FB). One of the interviewers (ALC) is a physician and researcher on topics revolving around access to care in low-resource settings. Input from the advocacy manager of an NGO that has been working in Afghanistan since 1999 (FB) together with the continuous feedback from Afghan NGO professionals throughout the study gave the team a deep understanding of the setting to ensure that data was analyzed as objectively as possible. No patient or public involvement occurred in this study.

### Data collection

An interview guide (Additional File 1) was developed following a timeline/storytelling approach so that interviewees would feel comfortable answering questions about the changes in access to care without explicitly referencing the change in government. The interview guide consisted of leading/probing questions arranged around three themes: changes/challenges in healthcare provision after August 2021; barriers faced by Afghans when accessing care; and recommendations to improve access to care for the population. The interview guide was piloted on a pool of researchers and refined in order to best obtain the relevant data.

Interviews lasted up to 1.5 h each and occurred during September and October of 2022. Upon consent, interviews were audio-recorded. All but three interviewees provided consent for the recording. Manual notes were also taken. Interviews were conducted in English. When the interviewee was not fluent in English, a local interpreter was recruited. Interviews with the NGO professionals took place at the organisation’s facilities, while those with hospital directors working for the MoPH were conducted in government hospitals or at the NGO hospital in Kabul. During the interviews with the hospital directors, a local EMERGENCY staff member facilitated the interaction between researchers and interviewees.

### Analysis and reporting

Interviews were transcribed using Sonix software (2023 Sonix, Inc.). Transcripts were manually checked to assess completeness and quality. A codebook was developed according to the study objective and used to code all transcripts, keeping it flexible to be adapted in case new codes emerged inductively from the text. Following a thematic analytic approach [23], codes were grouped into broader categories organized in accordance with the UHC framework by Boerma (2014) [24]. Barriers to access to care were conceptualised as three axes of a cube: coverage of health services (i.e., availability and quality of services), coverage of financial protection, and coverage of population. The analysis was performed by ALC, MV, MT and discrepancies were resolved after discussion. Data coding and analysis were performed using Atlas.ti software (version 22.2.0).

## Results

A total of 43 participants were interviewed. Eleven participants were representatives of government hospitals located across nine provinces. The remaining 32 participants were staff working for the EMERGENCY NGO (Table 1); 10 out of 43 participants were female.

**Table 1:** Study participants. FAP: first aid posts (stabilization and referral of emergency cases; PHC: primary health care centres)

	EMPLOYER	GENDER	PROFESSION	TYPE OF FACILITY	PROVINCE
1	Government	M	MD	Hospital	Kabul
2	Government	F	MD	Hospital	Kabul
3	Government	M	MD	Hospital	Kabul
4	NGO	M	Nurse	FAP	Kabul
5	NGO	M	MD	FAP	Kabul
6	NGO	M	Nurse	FAP	Parwan
7	NGO	M	Nurse	FAP/PHC	Logar
8	NGO	M	Pharmacist	FAP/PHC	Kabul
9	NGO	M	MD	FAP	Ghazni
10	Government	M	MD	Hospital	Wardak
11	NGO	M	Nurse	Hospital	Kabul
12	Government	M	MD	Hospital	Paktia
13	Government	M	MD	Hospital	Logar
14	NGO	F	Nurse	Hospital	Kabul
15	NGO	F	Nurse	Hospital	Kabul
16	NGO	M	Nurse	Hospital	Kabul
17	NGO	M	MD	Hospital - FAP	Panjshir
18	NGO	M	Nurse	Hospital	Panjshir
19	NGO	M	MD	Hospital	Panjshir
20	NGO	F	MD	Hospital	Panjshir
21	NGO	F	Midwife	FAP	Kapisa
22	Government	M	MD	Hospital	Kapisa
23	NGO	M	Nurse	FAP	Kapisa
24	Government	M	MD	Hospital	Parwan
25	Government	M	MD	Hospital	Panjshir
26	NGO	M	MD	Hospital	Panjshir
27	NGO	F	Midwife	Hospital	Panjshir
28	NGO	M	Nurse	Hospital	Panjshir
29	NGO	F	Nurse	Hospital	Panjshir
30	NGO	M	Nurse	Hospital	Helmand
31	NGO	M	Pharmacist	Hospital	Helmand
32	NGO	M	MD	Hospital	Helmand
33	NGO	M	Nurse	FAP	Helmand
34	NGO	F	Nurse	Hospital	Helmand
35	NGO	M	Nurse	FAP	Helmand
36	Government	M	MD	Hospital	Helmand
37	NGO	F	Nurse	Hospital	Helmand
38	NGO	M	Nurse	Hospital	Helmand
39	NGO	M	MD	Hospital	Helmand
40	NGO	M	Physiotherapist	Hospital	Helmand
41	NGO	F	MD	Hospital	Helmand
42	Government	M	MD	Hospital	Ghazni
43	NGO	M	MD	Hospital	Helmand

First, an overview of the reported post-August 2021 socio-political and epidemiological changes will be presented. Then, the results will be categorised according to the UHC framework: (1) coverage of health services; (2) coverage of financial protection; (3) coverage of population.

### Socio-political changes

The improved security conditions have led to an increase in internal mobility, allowing people easier access to urban areas. The city of Lashkargah in the Helmand province saw the most dramatic decrease in fighting, with roads reopening after having been battlefields for years.

In the past, if someone wanted to come to Lashkargah from other districts, they had to pass two, three battlefields. People were scared because of battlefields and mines. Now everyone is able to come directly to hospitals in Lashkargah.

(HCW, Helmand)

Unlike other parts of the country, Panjshir has seen an uptick in violence following August 2021 leading to a decrease in mobility, especially at night.

### Health profile

There has been a substantial increase in the number of patients visiting government clinics after August 2021. Three reasons were given for this increase: (1) the improved security situation; (2) the worsened financial situation made the cost of visiting private facilities excessive for many people; (3) the closure of many NGO clinics caused an overcrowding of public health facilities. Only health facilities in the Panjshir province, where episodes of fighting continue, have recorded a decrease in the number of patients following August 2021.

The pattern of diseases presenting at health facilities has changed after August 2021, with a substantial reduction in the number of war-wounded patients and a surge in civilian trauma (i.e., traffic accidents, falls from high surfaces).

*“Road traffic accidents have increased because there is no traffic law. Everyone comes from the villages by motorcycle or car and doesn’t know the traffic law […] and this creates a disaster when the person hits a car or bicycle.”*

(HCW, Ghazni)

Many participants reported an increase in the number of patients presenting with signs of malnutrition. Similarly, there has been an increase in mental health presentations, specifically for depression and anxiety. Though mental health problems were widespread even before August 2021, the compounding problems of poverty and unemployment have made them more apparent.

### Coverage of health services

#### Availability of health services

Afghanistan continues to experience a serious lack of resources at every level of health provision.

There are severe shortages of facilities, workers, equipment, and medications. Rural areas generally have fewer healthcare facilities than cities. Even when health facilities are present, they often have inadequate or insufficient resources. Many of the hospital directors raised issues over the current package of essential services, claiming that the existing guidelines rely on statistics that are not representative of the current context.

The resources that health facilities are given serve 1.5 million people, but in reality there are almost 4 million people living here now.

(*HCW, Helmand)*

There is a massive shortage of HCWs of all categories in public and private facilities, especially in rural districts, which has reportedly led to the closure of many hospitals. There are shortages of surgeons (i.e., orthopaedists, neurosurgeons, gynaecologists), radiologists, paediatricians, and anesthesiologists.

Everything deteriorated in the last year. We lost […] our good doctors, good engineers. They left the country, a great loss for our country […] We lost 1 or 2 generations.

(HCW, Kabul)

Medical equipment, along with staff to install and repair it, is also in short supply. The number of ambulances is insufficient, fuel is oftentimes unavailable. In Parwan, only two out of four ambulances are active; in Helmand, every district hospital has only one available ambulance despite the high number of patients. The number of available beds is also an issue.

I remember visiting a children’s hospital and seeing three or four children per bed.

(HCW, Panjshir)

Some provincial hospitals do not have basic laboratory equipment. There is also a shortage of imaging devices and surgical technology (e.g., laparoscopy tools). One large maternal centre in Kabul had only one ultrasound machine. Hospitals in all of Kabul’s neighbouring provinces regularly refer all patients requiring CT scans to the capital city because of these shortages.

After August 2021, there have been disruptions in the availability of consumables and medicines. Many facilities maintain an adequate stock only through NGOs support. However, these stocks can run out over a period of a few days when there is an overload of patients. The lack of supplies is more pronounced in rural hospitals which often only have basic medications. The procurement of anaesthetics, intravenous fluids, and consumables (e.g., bandages) is particularly difficult. Procurement issues arise mainly due to government import regulations. Recently, imports of medication have been blocked or slowed to support local production of medication. However, locally produced pharmaceuticals are not abundant enough to meet demand.

The ongoing support of aid organisations and NGOs, which provide hospitals with supplies and cover HCWs’ salaries, has been essential for maintaining health services across Afghanistan. Concerns were raised regarding the contracting process of NGOs.

#### Quality of health services

There are serious concerns about the quality of HCW education, medications, health facilities’ infrastructure, and the management of illness.

HCWs have poor knowledge due to the low quality of training programs in the country and the impact of the COVID-19 pandemic. The new government’s decision to ban females from education after sixth grade will result in fewer educational opportunities for female staff, with some exceptions for mid-level practitioners. Substandard training has led to increased mistrust of HCWs working in government clinics. Conversely, a great deal of trust is placed in international NGOs on account of their staff superior training, perceived commitment, and friendlier behaviour.

The quality of available medications has been called into question. Several cases of allergic reactions were reported, and concerns were raised about the way that drugs are transported and stored.

When we import medications, they are not kept the way they should be… no specific temperature. Some medications stay at the border for two, three months, even for one year without temperature control.

(HCW, Kabul)

The size and condition of many government-run health facilities is inadequate. Most of the buildings were built over 40 years ago, in accordance with the needs of the population at that time.

Our hospital was built for 50 beds, but now it has 550. That is still not enough because the demand is so high. […] What used to be a corridor is now used as a maternity ward […].

(HCW, Helmand)

Other health facilities are in buildings that used to be private houses and are now being rented to the government. Therefore, water and sanitation systems are inadequate for health facility standards. Some facilities lack access to hot water and have only intermittent electricity. There have been increases in child mortality in winter due to the lack of heating in some paediatric wards. One participant recalled performing surgery using the only light from mobile phones on a hot summer night without a functioning ventilation system. Health authorities are reluctant to renovate such facilities because they are not government property.

The management of non-communicable diseases (NCDs) is challenging at government clinics. Knowledge and awareness of NCDs among HCWs is low, and little time is spent on health promotion. Common medications for NCDs are usually unavailable in provincial hospitals and must be purchased at private pharmacies. Consultations for NCDs are generally delivered at private hospitals upon patients’ payment.

Regarding emergency services, patients are transferred in ill-equipped, understaffed ambulances or not transferred at all due to lack of transportation. For example, in the event of head trauma, a patient may be transported from neighbouring provinces to tertiary hospitals in Kabul or to private clinics to perform a CT scan at the patient’s expenses. When ambulances are unavailable, patients must arrange transportation between facilities themselves. This lack of organisation in head trauma management leads to enormous delays in treatment. At times, preventive craniotomies are performed “by blind eyes” because a CT scan could not be obtained.

The lack of expertise or adequate infrastructure almost always make open surgery the only option, even for procedures where laparoscopy is indicated (e.g., kidney stones). Few government hospitals perform elective surgeries. Thus, patients must secure the funds to have procedures done at private facilities or just continue treating symptoms with medications, risking having to undergo emergency surgery if a condition worsens. When major surgery is performed, follow-up care is frequently sought at NGO clinics, because government clinics would charge for dressings or medications.

### Coverage of financial protection

Although hospital directors said that basic health services are free of charge in government hospitals, the other participants reported that patients may face out-of-pocket payments to access basic services such as child delivery or surgical interventions.

*“Just the bed and the doctor’s examination are free at the MoPH facilities. For all the rest you have to pay”*.

(HCW, Chark)

Due to the scarcity of supplies, patients may be asked to purchase consumables and medicines from private pharmacies. Some patients resort to selling their property or incurring debt to pay for health services. Alternatively, patients might delay care until a health condition becomes urgent. There are cases of gall-bladder inflammation, fractures, and wounds becoming more complex and risky because of delay in seeking care due to financial hardship. Some patients go directly to traditional healers or pharmacies to ask for remedies or medications without prescription.

We have pharmacists here working as doctors, giving medications to everybody. Patients ask for medications, which he gives, since they can’t go to hospitals or private clinics where they have to pay both for the doctor and for medications.

(HCW, Helmand)

HCWs face financial insecurity as their remuneration is considered quite low and they are often irregularly paid. Some participants reported that the salary paid at some NGO facilities is lower than that paid at government clinics. Publicly employed HCWs often take on extra jobs, frequently at private facilities.

### Coverage of population

Geographically hard-to-reach communities face the most barriers to receiving adequate access to quality care. The cost of transportation to health facilities still remains unaffordable for the majority of these communities.

When they bring the child, we ask the mother: ‘Why didn’t you bring the baby two days ago when he started feeling sick?’ And the mother says: It’s dangerous and we don’t have transport. Since last year the situation’s been bad for mothers in Kapisa, Parwan and Panjshir. But for Panjshir it’s the worst.

(HCW, Panjshir)

Women experience limited freedom in accessing quality care, given that they often require permission from their husbands to visit clinics. This, coupled with geographical barriers and economic constraints, prevents some women from receiving timely and adequate care. At times, pregnant women present at the end of their pregnancy without having had any antenatal consultation. Unfortunately, the quality of health services offered to women is often mediocre. Women’s rights have decreased under the new government, and their barriers to access to care have increased.

After the change of government, participants reported that discrimination against ethnic minorities has increased.

## Discussion

The aim of this study was to perform a qualitative investigation into the main access to care challenges in Afghanistan, and how these challenges have been influenced by the recent socio-political changes in the country, from the perspectives of a pool of HCWs and hospital directors in 10 provinces. Following Boerma’s approach [24], this study used the UHC framework’s three dimensions to explore how the barriers to access to care have changed after August 2021 and what are the current obstacles to achieving UHC.

The lack of HCWs has worsened since the change in government [20, 25], especially in rural, hard-to-reach areas, as many professionals left the country after August 2021. The lack of female HCWs is even more pronounced. The longstanding gender imbalance among HCWs [20] is expected to worsen given the current restrictions on education for women [26, 27]. Failing to invest in education, particularly for female professionals, will stifle the country’s economic growth and stability [28].

Adequate coverage of health services is impaired by HCWs’ lack of expertise and awareness of major NCDs [29, 30], which leads to underdiagnosis and poor disease management [31]. The burden of NCDs is escalating in Afghanistan. The 2010 Afghanistan Mortality Survey reports that NCDs are the cause of over 35% of mortality in the country [32] and this number could be even higher due to underdiagnosis. A study that considered the period 2008–2019 shows that the death rate caused by NCDs stands at 55% for females and 45% for males and that these figures are expected to increase up to 60% and 47,6% respectively by 2030 [30].

The country’s health system needs to implement a multi-sectoral approach to expand its service offerings and effectively address the current emergent burden of diseases: a solid NCDs surveillance system should be established and health facilities need to be equipped with adequate resources for diagnosis and treatment; best practices for HCW training on NCD management need to be instituted [29]; citizen-focused NCD prevention programs must be strengthened, especially for women [30]. Afghanistan is now in the early stages of its demographic transition [33] and, until now, NCDs have ranked low among government and donor priorities [30]. Given that the transition will become more evident in the years to come [32], resource allocation for NCD programs needs to be prioritised. A project where such considerations could be integrated to foster inclusion of NCD care in essential packages of services is the Afghanistan Health Emergency Response (HER) project, founded by the World Bank and implemented through UNICEF to increase utilisation and quality of essential health services in Afghanistan [32].

Quality trauma management needs to be included in the coverage of health services. Despite the reduction in active fighting in the country, the burden of trauma-related cases remains high, having shifted from war-wounded cases to civilian trauma [34]. Funds should continue to be allocated to trauma and surgical care to ensure quality treatment for these patients. Investing in surgical care has a knock-on effect on the entire health system, improving the level of care in all health sectors and increasing the structural capacity to handle more complicated medical challenges [35]. HCWs should be adequately trained in the management of emergency cases. Every level of care needs to be equipped and staffed so that basic trauma management can occur across the territory, preventing patient overload in larger hospitals. An adequate, effective referral system should be structured to guarantee quality care to all patients. Concurrently, efforts should be made to curb the burden of civilian trauma by investing in the prevention of civilian injuries through incentives in the use of protective equipment or road safety campaigns [36, 37].

The study findings confirm that a major challenge to the realisation of UHC in Afghanistan is the financial burden that patients must bear to access health services [38]. It is of the utmost importance that an alternative financing mechanism be proposed to minimise out-of-pocket-expenses [39]. A transformative agenda has been proposed as a pathway to UHC in fragile humanitarian contexts where sustainable health financing can be reached through insurance systems funded by international support and progressive taxation [40]. Humanitarian aid is still necessary for providing basic healthcare in the country. Nevertheless, aid does not address chronic, structural deficits in the health system. Results from this study show that NGO-provided services are sometimes uncoordinated and impede the integration of the health system [41]. Any reform should prioritise universal access, avoiding duplication or lack of services in certain regions. It should take into account the view of all stakeholders, including the private sector and affected communities [42].

Lastly, the study confirms the extreme inequality that exists in terms of access to care. Women face specific obstacles when seeking care, and have been oppressed in all spheres of public life, including education [43, 44]. Large portions of Afghanistan’s rural population still have no access to basic care services [45]. Ethnic minorities are systematically discriminated against and denied access to basic services [46]. A commitment to equity is at the core of UHC [24] and no progress can be made if segments of the population continue to be left behind. Afghanistan’s future depends on a mutual commitment: authorities have to engage with the international community [42] and create a more representative, inclusive government, while the international community has to keep Afghanistan high on its agenda and commit to the country’s political, economic and social stability through a constructive engagement with authorities.

Afghanistan is now undergoing a post-conflict recovery phase. Scholars have highlighted several factors, such as the consensus on the need for UHC [47] and the well-established PHC system [48], that make this a unique opportunity for the country to revise its health system with effective strategies and interventions aimed at reaching UHC. Although UHC is typically assessed by measuring quantitative indicators, it is only by complementing those measurements with qualitative data that we can get an accurate picture of what is happening on the ground and what actions must be taken to move toward UHC [41, 49].

Finally, this study has limitations. First, it must be underlined that the findings represent the perspectives of a limited number of stakeholders, hence findings cannot be generalised to the entirety of Afghan health professionals. Similarly, the challenges identified by the interviewee of this study may not apply to the entire Afghan population. A total of thirty-two out of 43 interviews were conducted with NGO’s local staff, which prevents the results from being generalised to all HCWs. Reliance on local interpreters may have been a source of bias, although they were trained to report information as transparently as possible. Some participants may have been reluctant to share information that could be considered sensitive. Nevertheless, given Afghanistan’s peculiar situation, these results are valuable, and contribute to addressing the paucity of qualitative studies in fragile settings.

## Conclusions

Areas in Afghanistan that were inaccessible over the past decades due to conflict have now become reachable, offering a historical opportunity to hear Afghan voices. Health professionals and hospital directors are an invaluable source of information about access to care challenges. Interviews shed light on several weaknesses in the Afghan health system and highlighted chronic issues and issues that have deteriorated as a result of the socio-political changes in the country.

### Electronic supplementary material

Below is the link to the electronic supplementary material.

Supplementary Material 1

## Data Availability

The datasets used and analysed during the current study are available from the corresponding author on reasonable request.

## Abbreviations

UHC: Universal Health Coverage
WHO: World Health Organization
PHC: Primary Health Care
HCWs: Health Care Workers
COREQ: Consolidated Criteria for Reporting Qualitative Research
NGO: Non-governmental Organization
CRIMEDIM: Centre for Research and Training in Global Health, Humanitarian Aid, and Disaster Medicine
MoPH: Ministry of Public Health
NCDs: Non-communicable diseases
