# Supplementary material for: Exploring barriers to access to care following the 2021 socio-political changes in Afghanistan: a qualitative study
Source: Confl Health. 2024 Apr 24;18:36. doi: 10.1186/s13031-024-00595-4 (PMC11044283; doi:10.1186/s13031-024-00595-4)
Supplement: Supplementary file 1 — Supplementary Material 1 [file 13031_2024_595_MOESM1_ESM.docx]

**Additional file 1: Interview guide**

1. **What is your profession and role within [EMERGENCY NGO/health system] in Afghanistan?**
   1. What is your name? (depending on consent)
   2. In which facility do you work? (depending on consent)
   3. How was it like for you to become a [profession]?
      1. When did you decide to become a [profession]?
   4. Why did you decide to become a [profession]?
   5. When did you start working as a [profession]?
   6. Have you always been working in Afghanistan?
      1. If not, where did you work before?
      2. When did you start working as a [profession] in Afghanistan?
      3. Have you worked with other international organizations and/or NGOs?
   7. How has your work been impacted by historical events in Afghanistan?
      1. How was it like to provide care in Afghanistan back in [year he/she started working in Afghanistan]?
      2. What has changed in the way you provide care to the Afghan population today as compared to [year he/she started working in Afghanistan]?
      3. What has changed in the last year/since August 2021?
   8. What are the main challenges that you experience at work today?
      1. What in terms of equipment/ materials/services/staff is missing in your health facility to provide adequate care (make examples such as blood bank, drug quality and materials provision)?
   9. According to you, what are the things that work best within the health system in Afghanistan?
2. **Are you aware of any challenges/problems that families face when accessing healthcare services?**
   1. If yes, what are these problems?
   2. How has the ability to access health services changed today compared to [year he/she started working in Afghanistan]?
      1. If it has changed, what are the reasons for such change?
   3. Did you find any differences in the groups/categories of individuals accessing the health system today compared to [year he/she started working in Afghanistan]?
   4. According to you, how has access to care changed in the last year/since August 2021?
   5. Is there a difference between emergency and elective services?
3. **In your opinion, what needs to happen to improve access to care for the Afghan population?**
   1. What can you do as a [profession] to improve the way Afghan people access the health system?
   2. What can Afghan people do to better access the health system?
   3. What can the international community (e.g., donors, international NGOs) do to improve the way Afghan people access the health system?
   4. What can the Afghan authorities do to improve access to healthcare?

- Anything to add?
- Thanks and greetings
